# Crystal structure of [Co(NH_3_)_6_][Co(CO)_4_]_2_

**DOI:** 10.1107/S2056989015020290

**Published:** 2015-10-31

**Authors:** Thomas G. Müller, Florian Kraus

**Affiliations:** a Anorganische Chemie, Fluorchemie, Fachbereich Chemie, Philipps-Universität Marburg, Hans-Meerwein-Strasse 4, 35032 Marburg, Germany

**Keywords:** crystal structure, cobalt carbon­yl, ammonia, hydrogen bonding

## Abstract

Hexaamminecobalt(II) bis­[tetra­carbonyl­cobaltate(-I)], [Co(NH_3_)_6_][Co(CO)_4_]_2_, was synthesized by reaction of liquid ammonia with Co_2_(CO)_8_. The Co^II^ atom is coordinated by six ammine ligands. The resulting polyhedron, the hexa­amminecobalt(II) cation, exhibits point group symmetry -3. The Co^-I^ atom is coordinated by four carbonyl ligands, leading to a tetra­carbonyl­cobaltate(−I) anion in the shape of a slightly distorted tetra­hedron, with point group symmetry 3. The crystal structure is related to that of high-pressure BaC_2_ (space group *R*-3*m*), with the [Co(NH_3_)_6_]^2+^ cations replacing the Ba sites and the [Co(CO)_4_]^−^ anions replacing the C sites. N—H⋯O hydrogen bonds between cations and anions stabilize the structural set-up in the title compound.

## Abstract 2

The structure of hexa­amminecobalt(II) bis­[tetra­carbonyl­cobaltate(-I)] contains discrete octa­hedral [Co(NH_3_)_6_]^2+^ cations and [Co(CO)_4_]^−^ anions held together by N—H⋯O hydrogen bonds.

## Chemical context   

The reaction of Co_2_(CO)_8_ with bases has already been described in the literature (Hieber *et al.*, 1960 ▸). In addition, the reaction of dicobalt octa­carbonyl with liquid ammonia has been known for several decades (Behrens & Wakamatsu, 1966 ▸). Thereby Co_2_(CO)_8_ forms with NH_3_ hexa­ammine­cobalt(II) bis­[tetra­carbonyl­cobaltate(–I)], [Co(NH_3_)_6_][Co(CO)_4_]_2_, which is obtained as orange air-sensitive crystals. During this reaction, CO is released and reacts with ammonia to urea. However, structural data of of the title compound were missing and are presented in this communication.

## Structural commentary   

The cobalt atom Co1 of the hexa­amminecobalt(II) cation occupies Wyckoff position 3*a* with site symmetry 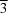.. It is coordinated by six symmetry-related ammine ligands in form of a slightly distorted octa­hedron. The Co—N distance in the [Co(NH_3_)_6_] octa­hedron is 2.1876 (16) Å which compares well with those of other reported hexa­amminecobalt(II) structures (Barnet *et al.*, 1966 ▸).

The cobalt atom Co2 of the tetra­carbonyl­cobaltate(–I) anion occupies Wyckoff position 6*c* and exhibits site symmetry 3.. It is coordinated by four carbonyl ligands in a shape close to an ideal tetra­hedron. The distances between the Co2 atom and the carbon atoms C1 and C2 of the ligands are 1.7664 (18) and 1.779 (3) Å, respectively. In the literature, distances in the range from 1.77 (2) to 1.82 (2) Å are reported for Co—C in the compound Co_2_(CO)_8_ (Sumner *et al.*, 1964 ▸). In the carbonyl ligands, the observed distances are in the expected range with 1.153 (2) and 1.140 (4) Å for C1—O1 and C2—O2, respectively. For the compound Co_2_(CO)_8_ distances from 1.14 (2) to 1.33 (2) Å were reported (Sumner *et al.*, 1964 ▸).

The crystal structure of [Co(NH_3_)_6_][Co(CO)_4_]_2_ can be derived from the high-pressure rhombohedral phase of BaC_2_ (BaC_2_ -HP1, *R* 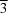 *m*) (Efthimiopoulos *et al.*, 2012 ▸). Formally, the Ba sites on Wyckoff position 3*a* are replaced by the hexa­ammine cobalt(II) octa­hedra and the C site on position 6*c* is replaced by the tetra­carbonyl­cobaltate(–I) tetra­hedron.

**Figure 1:**
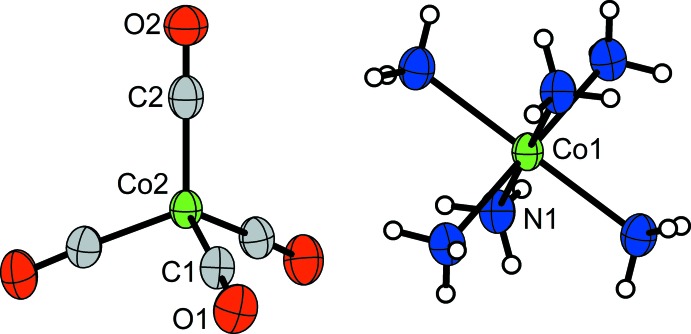
The mol­ecular structures of the tetra­carbonyl­cobaltate(−I) anion and of the hexa­amminecobalt(II) cation of the title compound. Displacement ellipsoids are shown at the 70% probability level. Labelling of symmetry-equivalent atoms has been omitted for clarity.

**Figure 2:**
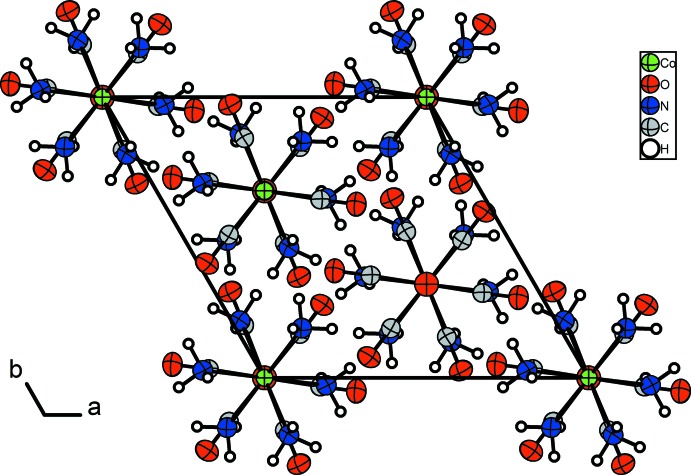
The unit cell of [Co(NH_3_)_6_][Co(CO)_4_]_2_, viewed along [001]. Displacement ellipsoids are shown at the 70% probability level.

The mol­ecular components of the title compound are shown in Fig. 1 ▸. The unit cell of [Co(NH_3_)_6_][Co(CO)_4_]_2_ projected along [001] is shown in Fig. 2 ▸.

## Supra­molecular features   

The arrangement of [Co(NH_3_)_6_]^2+^ octa­hedra and [Co(CO)_4_]^−^ tetra­hedra in the crystal structure is stabilized by N—H⋯O hydrogen bonds with the N1 atom as donor and the oxygen atoms O1 and O2 as acceptors atoms. One of the hydrogen bonds (N—H1*C*) is forked while, remarkably, in the neighbourhood of the hydrogen atom H1*B* no acceptor atom in the range of the sum of the van der Waals radii is present. Detailed information about hydrogen-bonding distances and angles are given in Table 1 ▸.

## Synthesis and crystallization   

**Figure 3:**
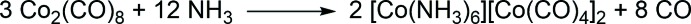
Reaction equation for the preparation of the title compound.

86 mg (29.4 mmol) of Co_2_(CO)_8_ were placed in a flame-dried bomb tube under argon. 0.2 ml of liquid ammonia were condensed to the bomb tube. The bomb tube, now containing an orange solution, was flame-sealed and stored at room temperature. The reaction equation is given in Fig. 3 ▸. After six months of crystallization time, moisture- and temperature-sensitive, orange single crystals of the title compound were obtained in almost qu­anti­tative yield from the still orange solution. After manual separation of the crystals under a light-optical microscope and evaporation of the solvent only a minute orange residue remained.

## Refinement   

Crystal data, data collection and structure refinement details are summarized in Table 2 ▸. All hydrogen atoms of the ammine ligands were located from a difference Fourier map and were refined isotropically without any further restraints.

## Supplementary Material

Crystal structure: contains datablock(s) I. DOI: 10.1107/S2056989015020290/wm5229sup1.cif

Structure factors: contains datablock(s) I. DOI: 10.1107/S2056989015020290/wm5229Isup2.hkl

CCDC reference: 1433399

Additional supporting information:  crystallographic information; 3D view; checkCIF report

## Figures and Tables

**Table 1:** Hydrogen-bond geometry (, )

*D*H*A*	*D*H	H*A*	*D* *A*	*D*H*A*
N1H1*A*O1^i^	0.87(4)	2.49(4)	3.159(2)	135(3)
N1H1*C*O1^ii^	0.87(3)	2.59(3)	3.290(2)	138(3)
N1H1*C*O2^iii^	0.87(3)	2.49(3)	3.249(3)	146(3)

Symmetry codes: (i) 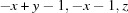; (ii) 
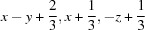
; (iii) 
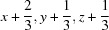
.

**Table 2:** Experimental details

Crystal data	
Chemical formula	[Co(NH_3_)_6_][Co(CO)_4_]_2_
*M*_r_	503.07
Crystal system, space group	Trigonal, *R* 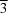
Temperature (K)	100
*a*, *c* ()	9.3679(4), 18.3089(18)
*V* (^3^)	1391.48(18)
*Z*	3
Radiation type	Mo *K*
(mm^1^)	2.70
Crystal size (mm)	0.16 0.12 0.08

Data collection	
Diffractometer	Stoe IPDS2T
Absorption correction	Integration (*X-RED32* and *X-SHAPE*; Stoe Cie, 2009 ▸)
*T*_min_, *T*_max_	0.649, 0.907
No. of measured, independent and observed [*I* > 2(*I*)] reflections	7025, 994, 910
*R*_int_	0.087
(sin /)_max_ (^1^)	0.724

Refinement	
*R*[*F* ^2^ > 2(*F* ^2^)], *wR*(*F* ^2^), *S*	0.034, 0.090, 1.08
No. of reflections	994
No. of parameters	52
H-atom treatment	All H-atom parameters refined
_max_, _min_ (e ^3^)	0.87, 0.65

Computer programs: *X-AREA* (Stoe Cie, 2011 ▸), *X-RED32* (Stoe Cie, 2009 ▸), *SHELXT* (Sheldrick, 2015*a* ▸), *SHELXLE* (Hbschle *et al.*, 2011 ▸) and *SHELXL2014* (Sheldrick, 2015*b* ▸), *DIAMOND* (Brandenburg, 2015 ▸) and *publCIF* (Westrip, 2010 ▸).

## supplementary crystallographic information

### Crystal data

**Table:**

[Co(NH_3_)_6_][Co(CO)_4_]_2_	*D*_x_ = 1.801 Mg m^−^^3^
*M**_r_* = 503.07	Mo *K*α radiation, λ = 0.71073 Å
Trigonal, *R*3	Cell parameters from 15618 reflections
*a* = 9.3679 (4) Å	θ = 3.3–33.4°
*c* = 18.3089 (18) Å	µ = 2.70 mm^−^^1^
*V* = 1391.48 (18) Å^3^	*T* = 100 K
*Z* = 3	Block, orange
*F*(000) = 759	0.16 × 0.12 × 0.08 mm

### Data collection

**Table:**

Stoe IPDS-2T diffractometer	994 independent reflections
Radiation source: sealed X-ray tube, 12 x 0.4 mm long-fine focus	910 reflections with *I* > 2σ(*I*)
Plane graphite monochromator	*R*_int_ = 0.087
Detector resolution: 6.67 pixels mm^-1^	θ_max_ = 31.0°, θ_min_ = 3.3°
rotation method scans	*h* = −13→13
Absorption correction: integration (*X-RED32* and *X-SHAPE*; Stoe & Cie, 2009)	*k* = −13→13
*T*_min_ = 0.649, *T*_max_ = 0.907	*l* = −26→26
7025 measured reflections	

### Refinement

**Table:**

Refinement on *F*^2^	Hydrogen site location: difference Fourier map
Least-squares matrix: full	All H-atom parameters refined
*R*[*F*^2^ > 2σ(*F*^2^)] = 0.034	*w* = 1/[σ^2^(*F*_o_^2^) + (0.0529*P*)^2^ + 1.0515*P*] where *P* = (*F*_o_^2^ + 2*F*_c_^2^)/3
*wR*(*F*^2^) = 0.090	(Δ/σ)_max_ < 0.001
*S* = 1.08	Δρ_max_ = 0.87 e Å^−^^3^
994 reflections	Δρ_min_ = −0.64 e Å^−^^3^
52 parameters	Extinction correction: *SHELXL2014* (Sheldrick, 2015), Fc^*^=kFc[1+0.001xFc^2^λ^3^/sin(2θ)]^-1/4^
0 restraints	Extinction coefficient: 0.0040 (8)

### Special details

**Table:**

Geometry. All e.s.d.'s (except the e.s.d. in the dihedral angle between two l.s. planes) are estimated using the full covariance matrix. The cell e.s.d.'s are taken into account individually in the estimation of e.s.d.'s in distances, angles and torsion angles; correlations between e.s.d.'s in cell parameters are only used when they are defined by crystal symmetry. An approximate (isotropic) treatment of cell e.s.d.'s is used for estimating e.s.d.'s involving l.s. planes.

### Fractional atomic coordinates and isotropic or equivalent isotropic displacement parameters (Å^2^)

**Table:**

	*x*	*y*	*z*	*U*_iso_*/*U*_eq_	
Co1	0.0000	0.0000	0.0000	0.01863 (18)	
Co2	−0.6667	−0.3333	0.04221 (2)	0.01972 (17)	
O1	−0.61903 (19)	−0.02591 (18)	0.10467 (9)	0.0315 (3)	
O2	−0.6667	−0.3333	−0.11725 (14)	0.0298 (5)	
N1	−0.0266 (2)	−0.2037 (2)	0.06820 (9)	0.0245 (3)	
C1	−0.6354 (2)	−0.1451 (2)	0.07846 (10)	0.0231 (3)	
C2	−0.6667	−0.3333	−0.05497 (19)	0.0237 (5)	
H1A	−0.121 (5)	−0.295 (5)	0.0656 (19)	0.054 (10)*	
H1B	0.034 (4)	−0.247 (4)	0.0558 (17)	0.038 (7)*	
H1C	−0.001 (4)	−0.176 (4)	0.1135 (19)	0.043 (8)*	

### Atomic displacement parameters (Å^2^)

**Table:**

	*U*^11^	*U*^22^	*U*^33^	*U*^12^	*U*^13^	*U*^23^
Co1	0.0160 (2)	0.0160 (2)	0.0240 (3)	0.00799 (10)	0.000	0.000
Co2	0.01726 (19)	0.01726 (19)	0.0247 (3)	0.00863 (9)	0.000	0.000
O1	0.0323 (7)	0.0236 (7)	0.0410 (8)	0.0158 (6)	−0.0025 (6)	−0.0036 (5)
O2	0.0316 (8)	0.0316 (8)	0.0262 (12)	0.0158 (4)	0.000	0.000
N1	0.0209 (7)	0.0212 (7)	0.0307 (7)	0.0101 (6)	−0.0002 (5)	0.0015 (5)
C1	0.0192 (7)	0.0203 (7)	0.0292 (8)	0.0095 (6)	−0.0007 (6)	0.0009 (6)
C2	0.0198 (8)	0.0198 (8)	0.0317 (15)	0.0099 (4)	0.000	0.000

### Geometric parameters (Å, º)

**Table:**

Co1—N1^i^	2.1876 (16)	Co2—C1	1.7664 (18)
Co1—N1^ii^	2.1876 (16)	Co2—C1^vi^	1.7664 (18)
Co1—N1^iii^	2.1876 (16)	Co2—C1^vii^	1.7664 (18)
Co1—N1^iv^	2.1876 (16)	Co2—C2	1.779 (3)
Co1—N1	2.1877 (16)	O1—C1	1.153 (2)
Co1—N1^v^	2.1877 (16)	O2—C2	1.140 (4)
			
N1^i^—Co1—N1^ii^	180.00 (9)	N1^iii^—Co1—N1^v^	90.65 (6)
N1^i^—Co1—N1^iii^	90.65 (6)	N1^iv^—Co1—N1^v^	89.35 (6)
N1^ii^—Co1—N1^iii^	89.35 (6)	N1—Co1—N1^v^	180.0
N1^i^—Co1—N1^iv^	89.35 (6)	C1—Co2—C1^vi^	106.76 (7)
N1^ii^—Co1—N1^iv^	90.65 (6)	C1—Co2—C1^vii^	106.75 (7)
N1^iii^—Co1—N1^iv^	180.00 (11)	C1^vi^—Co2—C1^vii^	106.75 (7)
N1^i^—Co1—N1	89.35 (6)	C1—Co2—C2	112.07 (6)
N1^ii^—Co1—N1	90.65 (6)	C1^vi^—Co2—C2	112.07 (6)
N1^iii^—Co1—N1	89.35 (6)	C1^vii^—Co2—C2	112.07 (6)
N1^iv^—Co1—N1	90.65 (6)	O1—C1—Co2	177.07 (17)
N1^i^—Co1—N1^v^	90.65 (6)	O2—C2—Co2	180.0
N1^ii^—Co1—N1^v^	89.35 (6)		

Symmetry codes: (i) *x*−*y*, *x*, −*z*; (ii) −*x*+*y*, −*x*, *z*; (iii) *y*, −*x*+*y*, −*z*; (iv) −*y*, *x*−*y*, *z*; (v) −*x*, −*y*, −*z*; (vi) −*y*−1, *x*−*y*, *z*; (vii) −*x*+*y*−1, −*x*−1, *z*.

### Hydrogen-bond geometry (Å, º)

**Table:**

*D*—H···*A*	*D*—H	H···*A*	*D*···*A*	*D*—H···*A*
N1—H1*A*···O1^vii^	0.87 (4)	2.49 (4)	3.159 (2)	135 (3)
N1—H1*C*···O1^viii^	0.87 (3)	2.59 (3)	3.290 (2)	138 (3)
N1—H1*C*···O2^ix^	0.87 (3)	2.49 (3)	3.249 (3)	146 (3)

Symmetry codes: (vii) −*x*+*y*−1, −*x*−1, *z*; (viii) *x*−*y*+2/3, *x*+1/3, −*z*+1/3; (ix) *x*+2/3, *y*+1/3, *z*+1/3.
